# Improving the effectiveness of ANT DBS therapy for epilepsy with optimal current targeting

**DOI:** 10.1002/epi4.12407

**Published:** 2020-08-09

**Authors:** Soila Järvenpää, Kai Lehtimäki, Sirpa Rainesalo, Timo Möttönen, Jukka Peltola

**Affiliations:** ^1^ Department of Neurosciences and Rehabilitation Tampere University Hospital Tampere Finland; ^2^ Faculty of Medicine and Health Technology Tampere University Tampere Finland

**Keywords:** 3D modeling, anterior nucleus of thalamus, deep brain stimulation, optimization, programming

## Abstract

**Objective:**

Deep brain stimulation of the ANT is a novel treatment option in refractory epilepsy with an established efficacy at the group level. However, data on the effect of individualized programming are currently lacking. We report the effect of programming changes on outcome in deep brain stimulation of anterior nucleus of thalamus (ANT DBS). Secondly, we investigated whether the effect differs between seizure types. Thirdly, we compared the response status between patients with stimulation contacts verified inside the ANT with patients with contacts located outside of ANT.

**Methods:**

The participants were 27 consecutive patients with ANT DBS implantation with at least two‐year follow‐up. Seizures were subdivided into focal aware (FAS), focal impaired awareness (FIAS), and focal to bilateral tonic‐clonic seizures (FBTCS). The patients’ seizure diaries were analyzed retrospectively to assess changes in different seizure types. Active contact locations for each patient were verified from preoperative MRI and postoperative CT fusion images using SureTune III (Medtronic Inc, Minneapolis, MN) software.

**Results:**

A significant reduction in monthly mean seizure frequency occurred in FIAS: 56% at two‐year and 65% at five‐year follow‐up. The effects on FAS and FBTCS were less pronounced. Patients with contacts inside the ANT or on the anterolateral border of ANT experienced a greater reduction in seizure frequency than patients with outside‐ANT contacts. Ultimately, seven patients became responders due to changes in DBS programming or repositioning of contacts, increasing our responder rate from 44% to 70% as measured by a seizure reduction of at least 50%.

**Significance:**

ANT DBS appears to be especially effective in reducing FIAS, when the appropriately chosen contacts are activated.

**Key points**

- Deep brain stimulation of anterior nucleus of thalamus for epilepsy is more effective with optimal current targeting
- Current can be targeted with stimulator programming or repositioning of contacts
- The greatest effect appears to be in reducing seizures with impaired awareness

## 1 INTRODUCTION

The effectiveness of deep brain stimulation (DBS) of the anterior nucleus of thalamus (ANT) in patients with refractory epilepsy was demonstrated in the SANTE trial, which had an initial randomized controlled double‐blind phase^1^ and an extended five‐year follow‐up period.^2^ Two different measures were evaluated at the end of the follow‐up of the SANTE trial. The mean decrease in the total number of seizures was 67%, and a similar percentage of patients experienced a greater than 50% reduction in seizure frequency. This improvement in efficacy with time is reminiscent of the situation with other forms of neuromodulation such as vagal nerve stimulation (VNS).^3^ The epilepsy type did not exert any major effect on the decrease in the numbers of seizures at the five‐year follow‐up. Therefore, SANTE demonstrated the long‐term efficacy of ANT DBS therapy but did not provide information about which patients would be the best candidates for this form of treatment.^2^ So far, the published data from single‐center studies have all adopted the same approach as applied in SANTE^1^ without providing data on the possibilities for optimizing treatment outcomes based on programming changes.^4^, ^5^, ^6^, ^7^, ^8^, ^9^, ^10^, ^11^, ^12^, ^13^, ^14^, ^15^

The ANT has been chosen as a stimulation target due to its location at a site that allows it to influence the brain's predisposition to epileptic seizures. The ANT is a major hub in the final common pathway of the spread of a seizure prior to impairment of consciousness and secondary generalizaton.^16^, ^17^, ^18^ In experimental studies, stimulation of the intralaminar nuclei of thalamus has exerted a major effect on consciousness, suggesting that DBS of thalamic structures may influence states with an impairment of consciousness or awareness.^18^

The results from previous studies on VNS imply that it might be rewarding to measure treatment outcomes in more wide‐ranging ways than only in terms of total seizure reduction. The data available from VNS point to a major reduction on seizure severity^3^ in addition to a decrease in the total number of seizures. Also, recent VNS studies have emphasized changes in the dominant seizure type as being the main parameter of efficacy.^19^

For these reasons, we set two distinct goals for this study. Firstly, we wanted to assess whether seizure outcomes could be improved by programming changes or reimplantation based on anatomy. We have recently demonstrated the importance of the detailed individualized anatomical knowledge of the stimulated contacts since these determine the probability that a patient will respond favorably^20^, ^21^ both in terms of seizure outcomes and in avoiding psychiatric side‐effects.^22^ Our second goal for this study was to analyze the effect of ANT DBS on specific seizure types with the emphasis on the impairment of consciousness.

## 2 MATERIALS AND METHODS

A total of 27 consecutive patients with ANT DBS with at least two years of follow‐up time were included in the study. The clinical features of the patients are presented in Table 1. The evaluation of etiology was based on magnetic resonance imaging (MRI) findings and clinical history. The seizure onset zone was assessed based on video‐EEG registrations.

**TABLE 1:** Patient demographics with respect to age, sex, years of education, etiology of epilepsy, MRI findings, localization of the epileptic zone by scalp EEG, antiepileptic drugs (AED), and outcome status defined by at least a 50% reduction in the seizure frequency

Patient	Age	Sex	Etiology	MRI	Epileptic zone	AED → at the end of follow‐up	Responder
1	28	M	Encephalitis	Normal	Multifocal	CLB 20 → 25, LTG 150, VPA 1500, ZNS 400	From the beginning
2	30	F	CD	Bilateral perisylvian polymicrogyria	Multifocal	CLB 15 → 30, ESL 0 → 2000, OXC 1500 → 0, ZNS 400 → 200	From the beginning
3	52	M	Encephalitis	Right parietal & temporal inflammatory lesion	Right temporal	CBZ 600 → 0, CLB 0 → 10, LCM 400 → 300	From the beginning
4	51	M	Unknown	Normal	Frontal	CBZ 400 → 0, CLB 15, ESL 0 → 1600, ZNS 400 → 500	From the beginning
5	34	M	CD	Bilateral periventricular heterotopia	Multifocal	CBZ 1000, CLB 20	From the beginning
6	35	M	CD	Bilateral periventricular heterotopia	Multifocal	CBZ 1200 → 0, LCM 0 → 600, CLB 30	From the beginning
7	29	M	Unknown	Normal	Right frontal	OXC 2700, PER 0 → 8, VPA 1300, ZNS 200 → 0	From the beginning^a^
8	39	F	CD	Hemimegalencephalia	Right frontal	CBZ 800 → 0, ESL 0 → 1600, LCM 200, LEV 1000, PER 0 → 12, ZNS 400	From the beginning
9	25	M	Unknown	Normal	Left parietal	CLB 30 → 20, ESL 0 → 1600, LEV 2000 → 0, OXC 1800 → 0, TPR 600 → 400	From the beginning^b^
10	24	M	Common variable immunodeficiency disease based autoimmune encephalitis	Signal enhancement in medulla oblongata and cerebellum	Multifocal	CLB 20, LCM 0 → 500, LEV 3000 → 2000, PER 0 → 10	From the beginning
11	32	M	Early venous infarct	Left temporal atrophy	Multifocal	LCM 400, LEV 3000 → 2000, LTG 400	From the beginning
12	35	F	Unknown	Normal	Right frontal	ESM 750, LCM 600, LEV 1000, ZNS 500	From the beginning
13	35	F	Encephalitis	Normal	Multifocal	CLB 20 → 25, LCM 0 → 450, OXC 1000 → 0, ZNS 500 → 300	After optimization
14	27	F	Encephalitis	Bilateral parietal inflammatory lesion	Multifocal	CLB 20 → 30, OXC 1500, PER 0 → 10, TPR 400 → 350	After optimization
15	45	M	CD	Bilateral polymicrogyric cortical dysplasia	Multifocal	CLB 10 → 25, OXC 2250, ZNS 400 → 300	After optimization
16	37	F	CD	Left frontal cortical dysplasia	Left frontal	CLN 8 → 6, LCM 0 → 500, PHT 200 → 0	After optimization
17	58	M	Unknown	Normal	Multifocal	OXC 1800	After optimization
18	54	M	Perinatal asphyxia	Right parietal and left occipital gliosis	Multifocal	CLB 10, LCM 400, PER 8 → 6	After optimization
19	52	F	Unknown	Normal	Multifocal	ESL 1600 → 0, LCM 0 → 400, PGB 600, ZNS 400 → 200	After optimization
20	22	M	Encephalitis	Normal	Multifocal	LCM 200, OXC 900, ZNS 500	Only for disabling seizures
21	25	M	CD	Bilateral subependymal periventricular heterotopia	Multifocal	CLB 30 → 20, OXC 1200, ZNS 200	No
22	27	M	Encephalitis	Normal	Multifocal	LCM 0 → 400, PER 0 → 10, VPA 2500 → 2000,	No
23	48	M	Unknown	Normal	Right frontal	CLB 0 → 5, LCM 400 → 500, LEV 3000 → 2000, OXC 1200 → 600,	No
24	51	M	CD	Bilateral perisylvian polymicrogyria	Right temporal	CLB 20 → 40, LCM 400, OXC 1500	No (dead)
25	57	F	CD	Left temporal sclerosis	Left temporal	LCM 500 → 600, TPR 400 → 200	No
26	34	F	Encephalitis	Left inflammatory lesion	Multifocal	ESL 2000, LEV 3000, ZNS 400	No
27	39	M	Unknown	Normal	Right frontotemporal	LEV 3000, LTG 200, VPA 900, ZNS 300	No

Note

AED changes have been reported at baseline and at the end of follow‐up, which was either 2 or 5 years.

Abbreviations: CBZ, carbamazepine; CD, cortical dysplasia; CLB, clobazam; CLN, clonazepam; ESL, eslicarbazepine acetate; ESM, ethosuximide; LCM, lacosamide; LEV, levetiracetam; LTG, lamotrigine; OXC, oxcarbazepine; PER, perampanel; PGB, pregabalin; PHT, phenytoin; TPR, topiramate; VPA, valproate; ZNS, zonisamide.

^a^ Fluctuation between periods of seizure freedom and relapse as described by Brodie et al (2012)[24].

^b^ Seizure frequency decreased > 50% but the overall situation remained suboptimal and the patient eventually died of epilepsy.

ANT DBS surgery was performed as reported previously.^21^ Stimulation was initiated on the fifth postoperative day. The initial stimulation parameters were cycles of 1‐minute on and 5‐minute off, 140 Hz frequency, and 90 µs pulse width. The amplitude was increased to 5 V during the first weeks of stimulation. Programming was adjusted according to individual needs. When different contacts were programmed, these were typically single contact cathodal with respect to the case as anode. If a second contact was added, the system became multicathodal. Bipolar stimulation was also tested in some of the patients.

The location of the DBS contacts was verified with SureTune III (Medtronic Inc) software using preoperative 3 Tesla MRI and postoperative CT. Contacts located inside the ANT or on the anterolateral border of the ANT but not below it (representing the thalamo‐cortical network) were deemed to have been successfully implanted. All other contacts were designated as being placed outside of the ANT.

Before the decision to operate, the seizure diaries of patients were carefully evaluated for reliability based on previous video‐EEG studies taking into account each patient's ability to remember and count seizures. The seizures were classified according to new ILAE classification^23^ into focal aware seizures (FAS), focal impaired awareness seizures (FIAS), and focal to bilateral tonic‐clonic seizures (FBTCS). Special attention was directed to the severity and duration of impaired awareness during the FIAS. For practical purposes, the duration of FIAS was categorized into very short seizures lasting less than 30 seconds and typical ones lasting more than 30 seconds. By its nature, this categorization is somewhat imprecise and arbitrary, but was based on a very early observation by the patients and their families in our center that the duration of period with impaired awareness seemed to decrease after the initiation of successful stimulation. After this preliminary observation, the patients and their caregivers were soon trained to prospectively monitor not only the seizure types, but also the duration of FIAS prior to the implantation to establish a more meaningful baseline seizure assessment.

The follow‐up visits were conducted by a single experienced epileptologist (JP) except for the first postimplantation visit which was undertaken by the neurosurgeon (KL).

The patients were classified as responders if there was ≥50% reduction in the seizure frequency occurring in the past 6 months as compared to baseline. Since defining a responder to epilepsy treatment is complicated and the artificial division into two groups does not always provide the most realistic impression of outcome,^24^ response status was further categorized into six different classes by total seizure frequency and disabling seizure frequency: Class 0 for seizure reduction of less than 25%, Class 1 for 25%‐50% seizure reduction, Class 2 for 50%‐75% seizure reduction, Class 3 for 75%‐90% seizure reduction, Class 4 for seizure reduction of more than 90%, and Class 5 for seizure freedom.

Wilcoxon signed‐rank test was used for statistical significance as a nonparametric test for related samples. The Bonferroni correction was used to adjust probability (p) value by multiplying the p value by the number of reviewed time points for which the seizure reduction rate was calculated.

The study was approved by the Ethics Committee of the Pirkanmaa hospital district. Written informed consent was obtained from each of the patients.

## 3 RESULTS

During the follow‐up visits, programming changes were made according to standard clinical practice. During the fall of 2013, individualized contact location information was available.^20^ The majority (n = 18) of patients had contacts within ANT or on the anterolateral aspect of ANT during the whole follow‐up period. In eight patients, the active contacts were not from the beginning in the ANT, and in five of these individuals, the contacts were changed successfully to those within ANT either by reprogramming or by reimplantation. Two of these eight patients (Patients 9 and 24) died of SUDEP before undergoing reimplantation, but this was not considered stimulation related because these patients had very frequent convulsive seizures already prior to initiation of neurostimulation therapy. They still suffered from a high frequency of disabling seizures even after seizure reduction accomplished by DBS. One patient with one unilateral contact in the ANT refused suggested reimplantation of the other, too inferiorly implanted lead (Patient 5). The contact location information was not available for one patient with cardiac pacemaker due to lack of high‐resolution MRI.

Prior to the recognition of the major effect of contact location, changes to voltage, pulse frequency, pulse duration, and cycling had been evaluated without any effect on seizure frequency. After gathering detailed contact location information, more than one contact was activated in several patients. In addition, parameter changes were made due to stimulation induced side‐effects in some patients. Table 2 describes the detailed information on programming changes.

**TABLE 2:**
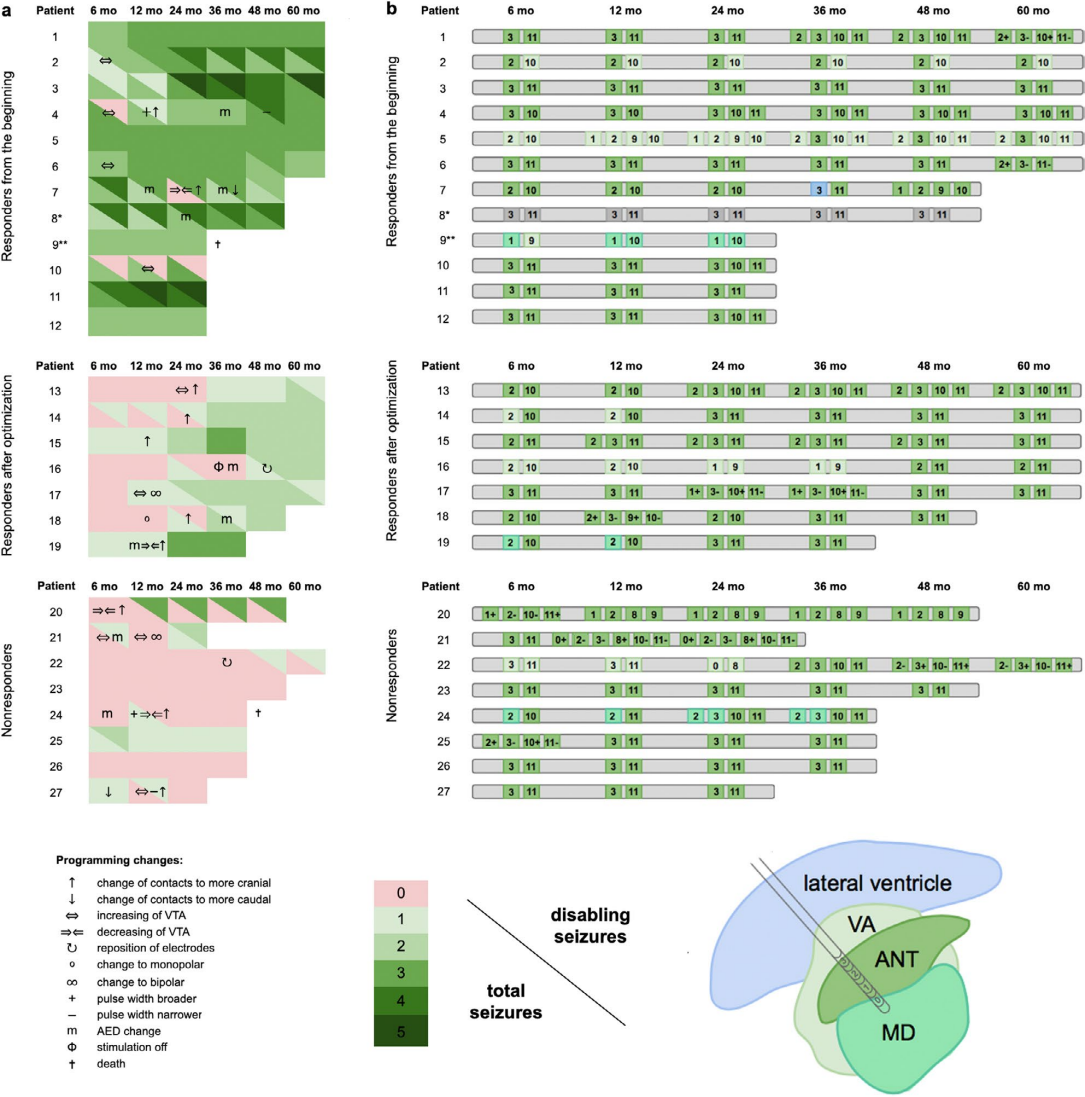
Relative change in seizure frequency in individual patients (A). Seizure categories are classified as follows: 0, seizure reduction <25%; 1, seizure reduction 25%‐50%; 2, seizure reduction 50%‐75%; 3, seizure reduction 75%‐90%; 4, seizure reduction ≥90%; and 5, seizure‐free. Class as assessed by total seizures is reported down on the left, class according to disabling seizure on the right. The changes in programming, electrodes, or AEDs leading to a change of class are depicted in symbols. Patient 8 (marked with an asterisk), despite enjoying a promising seizure reduction rate, later needed to have the implants removed due to an infection. Patient 9 (marked with two asterisks) displayed an over 50% seizure reduction in the most severe and predominant seizure type FBTCS; however, this had little effect on the complete picture: The number of seizures remained high, and this patient later died from epilepsy. Patients 20 and 21 were responders for disabling seizures but not for total seizures. Stimulated contacts in each patient (B). In Medtronic 3389 electrode, contacts 0‐3 are located on the left and contacts 8‐11 are on the right. Higher numbers are directed superiorly. Contacts in ANT are depicted in medium green, and contacts in VA are in light green and contacts in MD in turquoise. One contact was in lateral ventricle and is depicted in light blue. Patient 8 had cardiac pacemaker and lacks the location information due to a low‐quality MRI. Conversion from a nonresponder to a responder is marked with a red stripe

**FIGURE 1:**
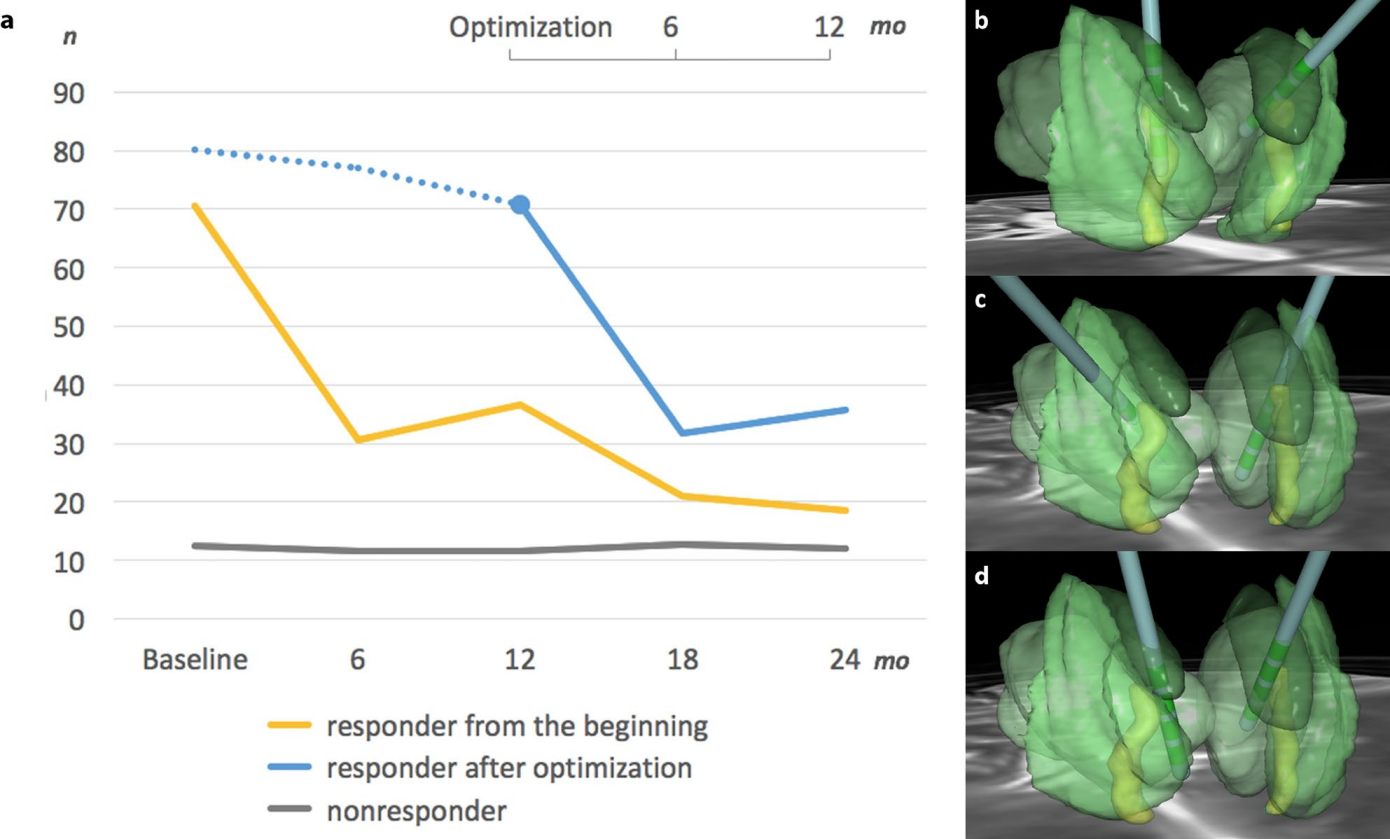
Total seizure reduction and the impact of exact contact location on seizure reduction at two‐year follow‐up (n = 27) (A). Mean monthly seizure frequency is depicted on the y‐axis. The seizure reduction in patients that were responders from the beginning (n = 12) is depicted in yellow and in patients that were nonresponders during the follow‐up (n = 8) are shown in gray (lower x‐axis). The seizure reduction in those patients who were initially nonresponders but later both contacts were optimized successfully (n = 7) is depicted in blue: The first 12 mo with no bilateral contacts in ANT are illustrated with a dotted line (lower x‐axis) and the first 12 mo in the same patients after optimization with a solid line (upper x‐axis). Examples of contact locations (B‐D). One patient with optimal contacts from the very beginning who responded well to treatment (B). A nonresponder with suboptimal contacts (C) later had the contacts optimized and changed to become a responder (D)

The relative change in the mean monthly number of seizures could be seen already during the first six months of treatment with DBS (Figure 1A). Seven patients had a statistically significant (*P* = .028) decrease in seizure rate of −12% at one‐year follow‐up but they only met the responder criteria later after optimization with a significant mean seizure reduction of −55% (*P* = .018). Examples of those patients with either optimal contacts or suboptimal but later optimized contacts are presented in Figure 1B–D.

If the stimulation site was in the ANT from the very beginning, there was a better response. The importance of the stimulation location and optimization is demonstrated in Figure 1 and Table 2.

Of the 18 patients that had activated contacts bilaterally within ANT or on the anterolateral aspect of ANT during the whole follow‐up period, nine were responders from the beginning, three were responders after optimization, and six were nonresponders. Optimizing by programming was most often reached with activating contacts that were more superior and in the ANT. Among the eight patients without active contacts in the ANT from the beginning, it was possible to obtain an optimal anatomical contact by changing the active contacts in three patients (Patients 14, 15, and 19) who became responders. In the other two patients, a reimplantation was performed to obtain bilateral contacts in the ANT (Patients 16 and 22), but only Patient 16 became a responder. For the one patient with initially one unilateral contact active in ANT (Patient 5) who refused suggested reimplantation, the active contacts were changed to the most superior ones by reprogramming and this allowed the volume of activated tissue around the other contact (that itself remained inferior to ANT) to reach ANT. This patient was a responder from the beginning, but the programming changes alleviated the patients’ psychiatric adverse effects.^22^

**FIGURE 2:**
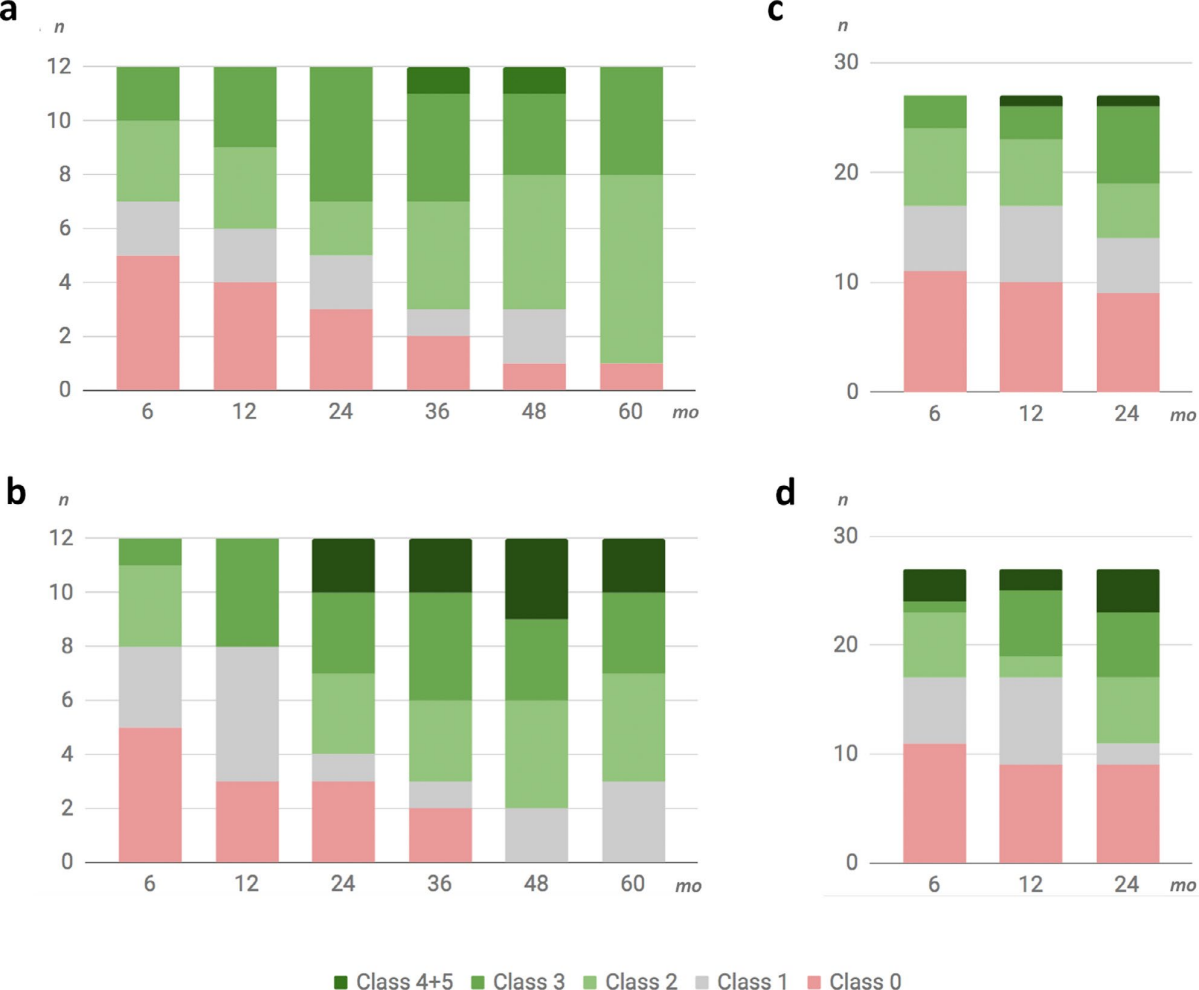
Reduction in total (A) and disabling seizures (B) in constant cohort^2^ of patients with a five‐year follow‐up (n = 12). Reduction in total (C) and disabling seizures (D) in constant cohort of patients with a two‐year follow‐up (n = 27). Seizure categories are classified as follows: 0, seizure reduction <25%; 1, seizure reduction 25%‐50%; 2, seizure reduction 50%‐75%; 3, seizure reduction 75%‐90%; 4, seizure reduction ≥90%; and 5, seizure‐free

The situation from the individual patient perspective is demonstrated in Table 2a showing the relative change with regard to different seizure types. The relative change in total and disabling seizures is represented in Figure 2, and the related optimization procedures in individual patients are displayed in Table 2.

**FIGURE 3:**
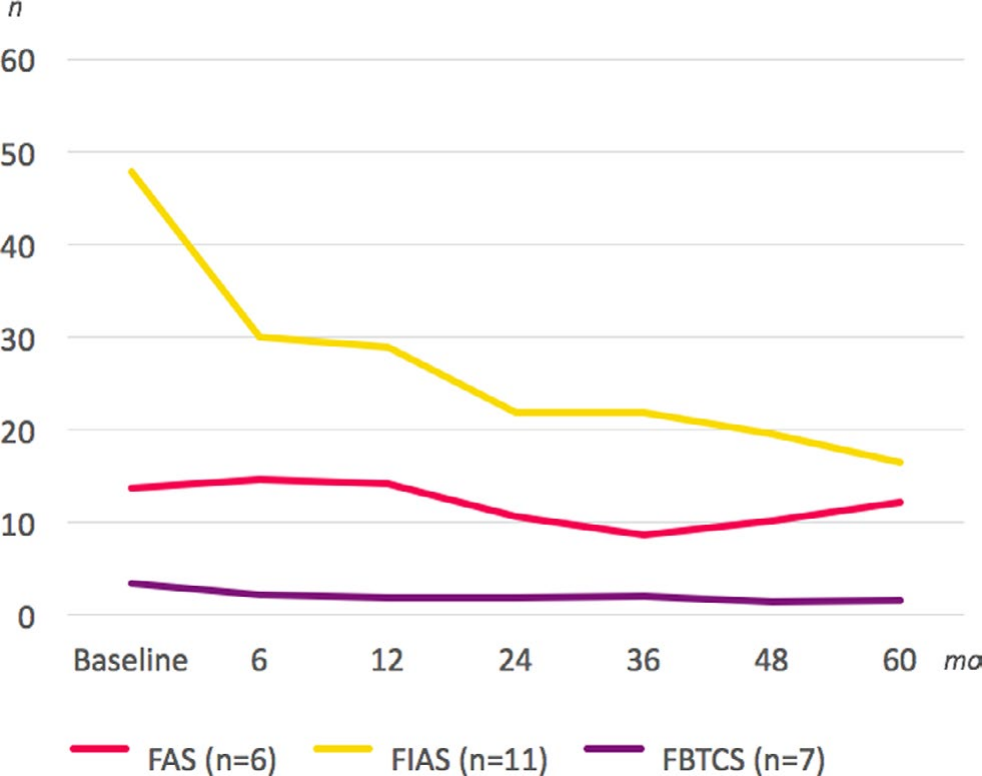
Mean seizure reduction in 5‐year follow‐up (n = 12): FAS, focal aware seizures (red); FIAS, focal impaired awareness seizures (yellow); and focal to bilateral tonic‐clonic seizures, FBTCS (violet)

When the seizure types were analyzed separately with regard to mean monthly seizure frequencies either for the first two years (Table 3a) or five years (Table 3b), differential effects could be discerned. The dominant seizure type was FIAS, which was present in 96% of the patients. FAS and FBTCS were both present in 56% of all patients. The most prominent change occurred in FIAS (56% reduction at two years (*P* = .009, n = 26) and 65% reduction at five years (*P* = .882, n = 12)). When FIAS were analyzed in terms of the duration of the impairment of awareness, the reduction in longer FIAS was 65% at two years (*P* = .003, n = 25) and 75% at five years (*P* = .066, n = 11), when the reduction in shorter FIAS was 41% at two years (*P* = 1.440, n = 12) and −56% at five years (*P* = .768, n = 7; Figure 3). When taken into account that for five patients, the duration of FIAS changed from the longer to the shorter type after initiation of ANT DBS, those seizures with an impairment of awareness for more than 30 seconds displayed a substantially larger effect than that encountered for very short seizures. The FBTCS rate reduction was 56% at two years (*P* = .069, n = 15) and 54% at five years (*P* = .108, n = 8). However, the mean pre‐DBS seizure frequency in FBTCS was only 4.5 per month.

**TABLE 3:** Two‐year cohort (a) and five‐year cohort (b)

(a)			
Seizure type	6 mo	12 mo	24 mo
FAS (n = 15)	−16% (*P* = .594)	−6% (*P *= 1.590)	−57% (*P* = **.033**)
FIAS total (n = 26)	−43% (*P* = **.045**)	−47% (*P *= **.003**)	−56% (*P* = **.009**)
FIAS < 30 s (n = 12)	−29% (*P* = 2.787)	−28% (*P* = 1.971)	−41% (*P* = 1.440)
FIAS> 30 s (n = 25)	−52% (*P* = **.021)**	−58% (*P* = **.003**)	−65% (*P *= **.003**)
FBTCS (n = 15)	−50% (*P* = .069)	−56% (*P* = .069)	−56% (*P* = .069)
Total	−37% (*P* = **.003**)	−37% (*P* = **.000**)	−57% (*P* = **.000**)

(b)						
Seizure type	6 mo	12 mo	24 mo	36 mo	48 mo	60 mo
FAS (n = 6)	7% (*P* = 4.116)	3% (*P* = 5.358)	−22% (*P* = .480)	−36% (*P* = .276)	−25% (*P* = 2.070)	−11% (*P* = 2.778)
FIAS total (n = 12)	−37% (*P* = .138)	−39% (*P* = .078)	−54% (*P* = .060)	−54% (*P* = **.048**)	−59% (*P* = **.048**)	−65% (*P* = .882)
FIAS < 30 s (n = 7)	−37% (*P* = 2.070)	−33% (*P* = 2.070)	−−51% (*P* = .768)	−53% (*P* = .696)	−49% (*P* = .696)	−56% (*P* = .768)
FIAS> 30 s (n = 11)	−38% (*P* = .126)	−46% (*P* = .108)	−57% (*P* = **.024**)	−55% (*P* = **.024**)	−69% (*P* = **.024**)	−75% (*P* = .066)
FBTCS (n = 8)	−36% (*P* = .168)	−45% (*P* = .168)	−47% (*P* = .168)	−42% (*P* = .126)	−58% (*P *= .072)	−54% (*P* = .108)
Total	−34% (*P* = .114)	−37% (*P* = **.018**)	−52% (*P* = **.048**)	−53% (*P *= **.030**)	−57% (*P* = **.018**)	−61% (*P* = **.036**)

Note

Relative change in different seizure categories in two‐year follow‐up (n = 27) and in five‐year follow‐up (n = 12). Significant *P* values are indicated with bold text.

FAS, focal aware seizure; FIAS < 30 s, focal impaired awareness seizure of duration less than 30 s; FIAS> 30 s, focal impaired awareness seizure of duration of at least 30 s; FBTCS, focal to bilateral tonic‐clonic seizure

Antiepileptic drugs (AEDs) were used as part of the treatment. There were two patients on monotherapy, six on two AEDs, 13 on three AEDs, and six on four AEDs at the time of initiation of ANT DBS. AED changes were made in 20 patients during the treatment. In two patients, the AED medication was increased; in three patients, it was decreased, and in 15 patients, some of their medications were increased, while some others were decreased. AED changes were made mainly due to tolerability issues. The AED changes led to increases in seizure frequency in two patients, while seizure control was not worsened in the other patients. In 13 patients, a new AED was initiated or the dose of preexisting AED increased but these changes did not lead to improvements in any patients. Six patients had their AED treatment unchanged during the follow‐up period. In one patient, stimulation was turned off because of an unclear situation (Patient 16), and later, PHT was withdrawn and LCM was initiated. This led to an improvement in seizure control that was less than 50% from the baseline. Only after repositioning of the DBS electrodes to the correct position, did this patient convert to a responder. In all of the other patients, AED changes were not responsible for the improvement in seizure control.

All individual programming changes are demonstrated in detail in the Table S1. 3D reconstructions of ANT and lead location together with therapy response are provided in the Figures S1–S3.

## 4 DISCUSSION

This study demonstrates the importance of individualized optimization of the stimulation parameters in patients with ANT DBS. These results highlight the crucial importance of the anatomical stimulation site as reported previously.^20^ The significance of stimulation location was demonstrated by the change in seizure outcomes when the optimal stimulation site was identified. Several patients were able to convert from nonresponder status to responder with optimal programming parameters. Our study has also a second major finding, that is the demonstration that the effect of ANT DBS treatment in our epilepsy patients is more pronounced in FIAS, where it seems to decrease both the frequency and duration of these seizures. Furthermore, the frequency of FBTCS also decreased, but the clinical significance was of lesser importance, since the majority of those who had frequent tonic‐clonic seizures before undergoing ANT DBS were still remarkably disabled by the amount of the remaining seizure burden. These two seizure categories were the dominant seizure types in our study. The effect was not dependent on the epilepsy type, and there was no difference with regard to the seizure onset zone.

Currently, there are only limited amount of data available regarding programming changes. In the long‐term phase of the SANTE study, changing stimulation voltage from 5.0 to 7.5 V or frequency from 145 to 185 Hz did not show an effect on the seizure frequency more than initial settings.^1^ We described previously patients that developed psychiatric adverse effects associated with ANT DBS programming settings.^22^ As new‐onset psychiatric symptoms are a common finding following epilepsy surgical procedures, these symptoms related to ANT DBS can be alleviated by changing the stimulation intensity and different active contacts. The adverse effects might have been due to off‐target stimulation given that the ANT has links to many neural circuits related to emotion and cognition.^25^ In addition to these, many of the previous studies regarding ANT DBS have concentrated on the optimal stimulation target, seizure reduction rate, and selecting a surgical trajectory. Instead, the effects of changing the stimulation site on outcomes have not been reported. In our current study, seven patients out of 27 became responders due to changes in DBS programming or repositioning of contacts. Reprogramming the DBS increased our responder rate from 44% to 70%, if this is measured by a seizure reduction of at least 50%. The present study highlights the need to carefully monitor the seizure changes in addition to the mental status of patients undergoing DBS implantation so that therapeutic adjustments can be made as necessary.^25^

Our study provides additional evidence about the importance of the exact anatomical locations of the contacts responsible for ANT DBS stimulation.^20^ After reprogramming (change of active contacts) or reimplantation, marked therapeutic effectiveness was observed. Choosing active contacts that were located more superior inside the ANT seemed to be beneficial. Furthermore, in several patients, the addition of a second active contact to widen the volume of tissue activated seemed to confer additional positive effects. Other changes to stimulation parameters such as changes in voltage, pulse width, stimulation frequency, or alteration of stimulation cycle were done occasionally, but the effect of these changes was minimal in comparison with the contact selection. Although the seizure reduction appears to improve over time, the reprogramming related improvements in seizure reduction occurred quickly during the next three months after changing the active contacts. In most of the previous studies,^1^, ^2^, ^4^, ^21^ the exact individual anatomic locations have not been detailed, making it difficult to draw reliable conclusions about the efficacy of ANT DBS stimulation. In one study, the combined activation maps from responding contacts were plotted on an atlas‐based ANT‐anatomy, suggesting that there would be a hot spot located in the inferior and lateral part of ANT in close proximity to the mammillothalamic tract.^26^ Unfortunately, this approach does not take into account the individual variations in the anatomy of the ANT.^20^, ^27^ Van Gompel et al (2015)^28^ also emphasized that the anterior nucleus seems to be a difficult location to reliably target and proposed that an alternative route via a posterior inferior parietal approach.

The most significant effect in our study was observed in patients with FIAS. In FBTCS, there was over 50% reduction in seizure rate in the whole group, but the results were distorted by two patients with initially 12‐25 monthly FBTCS who achieved significant decreases in the seizure frequency during ANT DBS treatment. The other patient died of SUDEP two years after ANT DBS implantation and was thus clinically considered as a nonresponder. The majority of patients in this group (12 out of 15) had three or less FBTCS per month before undergoing ANT DBS. Only six out of 15 patients had ≥50% reduction in FBTCS, and for one‐third, there was no reduction in the FBTCS rate. Therefore, the effects on FBTCS seemed less pronounced. Since FIAS and FBTCS were the predominant seizure types in most of our patients, these effects were most straightforward to quantify.

In the first SANTE analysis of the three‐month blinded phase, some information concerning the seizure types was obtained. The seizure type prospectively designated by the participant as being most debilitating improved by 40% in the stimulated group versus 20% in the control group. During the blinded phase, injuries produced by seizures occurred in 26% of the control subjects but only in 7% of the actively stimulated subjects, highlighting the impact on seizure severity.^1^ In the long‐term follow‐up, the median reduction for temporal lobe seizures amounted to 76%; for frontal lobe seizures, it was 59%. The remaining seizure onset locations displayed a median reduction of 68% at 5 years.^2^ These long‐term follow‐up data demonstrate the ANT DBS is also efficacious for other epilepsy types in addition to temporal lobe epilepsy. Nonetheless, it is frequently postulated that ANT DBS therapy is only effective in patients with limbic seizures.^29^ Similarly, in an 11‐year single‐center experience, no difference with respect to epilepsy types was observed, with a median 70% seizure reduction being reported.^12^ In one randomized controlled study and in several noncontrolled studies investigating ANT DBS, approximately every second patient with intractable epilepsy achieved good seizure reductions which ranged from 46% to 90%.^4^, ^5^, ^6^, ^7^, ^8^, ^9^, ^10^, ^11^, ^12^, ^13^, ^14^, ^15^ Our median seizure reduction for all seizures at five years is similar to previous studies.

To date, however, previous ANT DBS studies have not investigated whether neurostimulation might specifically restore an impaired level of consciousness when the patient is in ictal or postictal states. There are preclinical studies supporting the theory that activation of the intralaminar thalamic nuclei with neurostimulation would be a promising neurosurgical target for improving the level of consciousness during and after seizures.^18^ In an animal model, ANT DBS has been reported to induce parameter‐dependent activation within the temporal, prefrontal, and sensorimotor cortices.^30^ The anterior nucleus of thalamus has extensive connections to the anterior cingulate cortex, a structure modulating the default mode network (DMN), which is responsible for the preservation of consciousness. Therefore, ANT DBS stimulation may be involved in preventing the down‐regulation of DMN, which has been shown to occur during FIAS.^31^ Greater positive connectivity with DMN has been reported in ANT DBS responders as compared to nonresponders.^32^ In our study, more significant changes were observed in longer FIAS supporting the role of ANT DBS stimulation in restoring consciousness during seizures. Even though the duration of impaired awareness can be difficult to assess with precision, this aspect may be the first indications of positive effects of stimulation after the initiation of the therapy, highlighting the importance of paying attention to these details in the seizure diaries.

It is not straightforward to assess the seizure reduction since patients that are treated with ANT DBS are also receiving AEDs. Taking the patient's best interest as a priority, some AED changes usually have to be undertaken due to tolerability issues or in attempts to improve seizure control during long follow‐up periods. It can be debated whether it is the neurostimulation or the AEDs that are responsible for changes in seizure frequency.^33^ In our patient population, only one patient changed to another seizure reduction class after the AED medication was altered. This patient had the stimulator turned off before the AED change due to an unclear response to treatment and had a slight improvement in seizure frequency after the alteration of medication, but the significant change into a responder happened for this patient only later after reposition of the DBS contacts from a suboptimal location to ANT.

There were two people in our study that died of SUDEP not considered to be stimulation‐related. According to a recent systematic review, the clinical safety of ANT DBS is supported by several studies suggesting that the treatment is well tolerated among refractory epilepsy patients.^34^ In a recent case report of a patient who died of SUDEP eight months after undergoing ANT DBS surgery, a postmortem study showed only mild inflammation along the lead track and no significant microscopic or histochemical differences compared with a control tissue of an individual without epilepsy.^35^ In our center, patients that have been selected for ANT DBS treatment suffer from a drug‐resistant epilepsy with high frequency of disabling seizures and if epilepsy surgery or VNS have been inapplicable or unsuccessful. This might have caused a selection bias that might explain the relatively high amount of SUDEP in our study, as included has been patients with a significant seizure load over time, a matter that is often associated with cognitive decline and neuronal damage.^36^ These changes might lead to decreased connectivity between ANT and remote structures, which might impact on the effects of DBS.^37^ Thus, ANT DBS should not be used as a last resort therapy, but to be considered in the early stages of refractory epilepsy.

The limitations of our study include the small number of patients representing all of the different epilepsy and seizure types; in particular, we lacked patients with bitemporal epilepsy. Using Bonferroni correction comes at the cost of reducing statistical power, but in turn diminishes a chance of incorrectly rejecting a null hypothesis in a set‐up of multiple comparisons. In addition, the nonresponders had a lower seizure frequency before ongoing ANT DBS treatment compared to responders, which might have a distorting impact on some results. On the other hand, we did have exact details of the individualized anatomy of the stimulation sites combined with special attention to patient seizure diaries, highlighting the importance of reporting detailed anatomical data with reference to seizure and other outcomes.

## 5 CONCLUSIONS

The characterization of the response of ANT DBS on the different seizure types seems to be helpful in selecting those patients most suitable for this form of therapy, with our results indicating that the best treatment response is obtained for seizures with impaired awareness. The efficacy of this therapy can be significantly increased with optimal programming and contact location when the total seizure count is used as an outcome measure. In the future, we need to investigate other optimal programming parameters in addition to contact activation and to establish better criteria for selecting the most suitable candidates for different models of neurostimulation.^38^

## CONFLICT OF INTEREST

SJ has received study grants from Maire Taponen Foundation, Finnish Epilepsy Research Foundation, Finnish Medical Foundation and Instrumentarium Science Foundation. JP has received speaker and consultation fees from Medtronic. KL has received speaker honoraria and travel grant from Medtronic, and Abbot. KL has received lecture fees from Medtronic, Otsuka Pharmaceutical, and Lundbeck and has been sponsored to travel and attend to a medical congress by Medtronic. SR declares no conflict of interest. TM has received study grants from Finnish Epilepsy Research Foundation, Maire Taponen Foundation, and Finnish Medical Foundation and has been sponsored to travel and attend medical congresses by Medtronic and Boston Scientific. We confirm that we have read the Journal's position on issues involved in ethical publication and affirm that this report is consistent with those guidelines.

## AUTHOR CONTRIBUTIONS

All authors have made substantial contributions to the conception and design of the study, acquisition of data, analysis, and interpretation of data, drafting the article and revising it critically, and final approval of the version to be submitted.

## Supporting information

Supplementary Material
