# Supplementary material for: Improving the effectiveness of ANT DBS therapy for epilepsy with optimal current targeting
Source: Epilepsia Open. 2020 Aug 9;5(3):406–17. doi: 10.1002/epi4.12407 (PMC7469781; doi:10.1002/epi4.12407)
Supplement: Supplementary file 1 — Supplementary Material [file EPI4-5-406-s001.zip › epi412407-sup-0004-TableS1.1.docx]

**Supplementary material (S1)**

**Table S1.1.** Changes in stimulation sites, programming parameters and AEDs in individual patients during the follow-up. Other factors with possible effects on seizure frequency are also reported. The programming parameters refer to bilateral contacts unless indicated otherwise. Single contact numbers without a sign refer to monopolar, single contact cathoded with respect to the case as anode. Multiple contacts on one side refer to multi-cathodal system. Contact numbers with plus or minus signs indicate bipolar stimulation. Reposition means reposition of both DBS leads. Abbreviations: CBZ, carbamazepine; CLB, clobazam; ESL, eslicarbazepine acetate; Hz, Hertz; IPG, internal pulse generator; LCM, lacosamide; LTG, lamotrigine; OC, ocarbazepine; PER, perampanel; TPR, topiramate; V, volt; VPA, valproate; ZNS, zonisamide; μs, microsecond, * condition affecting physical health (i.e. subdural or subarachnoidal hemorrage after fall, pregnancy related conditions) or inducing psychic stress (i. e. divorce, accident of a relative); ^a^ stimulation turned off due to unclear situation; ^b^ non-intentional programming change

|  | |  | |  |  |  |  |  |  |  |
| --- | --- | --- | --- | --- | --- | --- | --- | --- | --- | --- |
| **Patient** | | | **0-3 mo** | | **6 mo** | **12 mo** | **24 mo** | **36 mo** | **48 mo** | **60 mo** |
| 1 | contacts | | 3 / 11 | |  |  | 2,3 / 10,11 |  | 1+,2-,3- / 9+,10-11- |  |
|  | voltage | | 5 / 5 V | |  | 6 / 6 V | 4 / 4 V |  | 5 / 4 V |  |
|  | pulse width | | 90 μs | |  |  | 120 μs |  | 90 μs |  |
|  | frequency | | 140 Hz | |  |  |  |  |  |  |
|  | cycling on / off | | 1 / 5 min | |  |  |  |  |  |  |
|  | other | |  | |  |  |  | IPG change |  |  |
| 2 | contacts | | 2 / 10 | |  |  |  |  |  |  |
|  | voltage | | 5 / 5 V | | 6.5 / 6.5 V |  |  |  |  |  |
|  | pulse width | | 90 μs | |  |  |  |  |  |  |
|  | frequency | | 140 Hz | |  |  |  |  |  |  |
|  | cycling on / off | | 1 / 5 min | |  |  |  |  |  |  |
|  | AED changes | |  | |  | ↑OC, -ZNS |  | ↑CLB |  |  |
|  | other | |  | |  |  |  | * | * IPG change |  |
| 3 | contacts | | 3 / 11 | |  |  |  |  |  |  |
|  | voltage | | 5 / 5 V | |  |  |  |  |  |  |
|  | pulse width | | 90 μs | |  |  |  |  |  |  |
|  | frequency | | 140 Hz | |  |  |  |  |  |  |
|  | cycling on / off | | 1 / 5 min | |  |  |  |  |  |  |
|  | AED changes | |  | |  | -CBZ, +CLB, ↓LCM |  |  |  |  |
|  | other | |  | |  |  |  |  |  |  |
| 4 | contacts | | 3 / 10 | |  | 3 / 10,11 |  |  |  |  |
|  | voltage | | 5 / 5 – 6 / 6 V | | 7 / 7 V | 5 / 5 V |  |  | 6 / 5 V |  |
|  | pulse width | | 90 μs | |  | 120 μs | 150 μs |  | 120 μs |  |
|  | frequency | | 140 Hz | |  |  |  |  |  |  |
|  | cycling on / off | | 1 / 5 min | |  |  |  |  |  |  |
|  | AED changes | |  | |  | ↑CBZ, +ZNS |  | -CBZ, +ESL |  |  |
|  | other | |  | |  |  |  |  |  |  |
| 5 | contacts | | 2 / 10 | | 1,2 / 9,10 |  | 2,3 / 10,11 |  |  |  |
|  | voltage | | 5 / 5 V - 6 / 6 V | | 6.5 / 6.5 V |  |  |  |  |  |
|  | pulse width | | 90 μs | |  |  |  |  |  |  |
|  | frequency | | 140 Hz | |  |  |  |  |  |  |
|  | cycling on / off | | 1 / 5 min | |  |  |  |  |  |  |
| 6 | contacts | | 2 / 10 – 3 / 11 | |  |  |  |  | 2+,3- / 11- |  |
|  | voltage | | 5 / 5 | | 6 / 6 V | 5 / 5 V |  |  |  |  |
|  | pulse width | | 90 μs | |  | 120 μs |  |  |  |  |
|  | frequency | | 140 – 180 Hz | |  |  |  |  |  |  |
|  | cycling on / off | | 1 / 5 min | |  |  |  |  |  |  |
|  | AED changes | |  | |  | IPG change |  |  |  |  |
| 7 | contacts | | 2 / 10 | |  |  | 3 / 11 | 1,2 / 9,10 |  |  |
|  | voltage | | 5 / 5 V | |  |  | 4.5 / 4.5 V | 4 / 4 V |  |  |
|  | pulse width | | 90 μs | |  |  |  |  |  |  |
|  | frequency | | 140 Hz | |  |  |  |  |  |  |
|  | cycling | | 1 / 5 min | |  |  |  |  |  |  |
|  | other | |  | |  | -ZNS, +PER | +ZNS | ↓PER |  |  |
| 8 | contacts | | 3 / 11 | |  |  |  |  |  |  |
|  | voltage | | 5 / 5 – 6 / 6 V | | 7 / 7 V |  |  |  |  |  |
|  | pulse width | | 90 μs | |  |  |  |  |  |  |
|  | frequency | | 140 Hz | |  |  |  |  |  |  |
|  | cycling on / off | | 1 / 5 min | |  |  |  |  |  |  |
|  | AED changes | |  | |  |  | +PER | -CBZ, +ESL |  |  |
| 9 | contacts | | 0 / 8 | | 1 / 9 | 1 / 10 |  |  |  |  |
|  | voltage | | 5 / 5 V | | 6 / 6 V | 5 / 5 V |  |  |  |  |
|  | pulse width | | 90 μs | |  | 180 μs |  |  |  |  |
|  | frequency | | 140 Hz | |  |  |  |  |  |  |
|  | cycling on / off | | 1 / 5 min | |  |  |  |  |  |  |
|  | AED changes | |  | |  |  |  |  |  |  |
| 10 | contacts | | 3 / 11 | |  |  | 3 / 10,11 |  |  |  |
|  | voltage | | 5 / 5 V | |  | 6 / 6 V |  |  |  |  |
|  | pulse width | | 90 μs | |  |  |  |  |  |  |
|  | frequency | | 140 Hz | |  |  |  |  |  |  |
|  | cycling on / off | | 1 / 5 min | |  |  |  |  |  |  |
| 11 | contacts | | 3 / 11 | |  |  |  |  |  |  |
|  | voltage | | 3.5 / 4 V | |  |  |  |  |  |  |
|  | pulse width | | 90 μs | |  |  |  |  |  |  |
|  | frequency | | 140 Hz | |  |  |  |  |  |  |
|  | cycling on / off | | 1 / 5 min | |  |  |  |  |  |  |
|  | AED changes | |  | | ↓LEV |  |  |  |  |  |
| 12 | contacts | | 3 / 11 | |  | 3 / 10,11 |  |  |  |  |
|  | voltage | | 3.5 / 4 – 5 / 5 V | |  | 5 / 4 V |  |  |  |  |
|  | pulse width | | 90 μs | |  |  |  |  |  |  |
|  | frequency | | 140 Hz | |  |  |  |  |  |  |
|  | cycling on / off | | 1 / 5 min | |  |  |  |  |  |  |
| 13 | contacts | | 2 / 10 | |  |  | 2,3 / 10,11 |  |  |  |
|  | voltage | | 5 / 5 – 6 / 6 V | | 7 / 7 V |  | 5 / 5 V |  |  |  |
|  | pulse width | | 60 - 90 μs | |  |  |  |  |  |  |
|  | frequency | | 140 Hz | |  |  |  |  |  |  |
|  | cycling on / off | | 1 / 5 min | |  |  |  |  |  |  |
|  | AED changes | |  | | +LCM, -OC, ↓CLB, ↓ZNS | ↑LCM |  |  |  |  |
|  | other | |  | |  |  | * | * |  |  |
| 14 | contacts | | 2 / 10 | |  |  | 3 / 11 |  |  |  |
|  | voltage | | 5 / 5 V | |  |  | 5 / 4 V |  |  |  |
|  | pulse width | | 90 μs | |  | 150 μs |  |  |  |  |
|  | frequency | | 140 Hz | |  |  |  |  |  |  |
|  | cycling on / off | | 1 / 5 min | |  |  |  |  |  |  |
|  | AED changes | |  | |  |  |  | +PER, ↓TPR |  |  |
| 15 | contacts | | 2 / 10 | | 2 / 11 | 2,3 / 11 |  |  | 3 / 11 |  |
|  | voltage | | 4 / 4 – 5 / 5 V | |  |  |  |  |  |  |
|  | pulse width | | 90 μs | |  |  |  |  |  |  |
|  | frequency | | 140 Hz | |  |  |  |  |  |  |
|  | cycling on / off | | 1 / 5 min | |  |  |  |  |  |  |
|  | AED changes | |  | |  |  |  | ↓ZNS | ↑ZNS |  |
| 16 | contacts | | 2 / 10 | |  | 1 / 9 |  |  | 2 / 11 |  |
|  | voltage | | 5 / 5 – 7 / 7 V | |  | 5 / 5 V | 4 / 4 |  | 2.5 – 2.5 V |  |
|  | pulse width | | 90 μs | |  |  | 150 μs |  | 90 μs |  |
|  | frequency | | 140 Hz | |  | 60 Hz | 140 Hz |  | 140 Hz |  |
|  | cycling on / off | | 1 / 5 – 1 / 3 min | |  | 1 / 5 min |  |  | 1 / 5 min |  |
|  | AED changes | |  | | ↓CLN |  | -PHT, +LCM |  |  |  |
|  | other | |  | | DBS off ^a^ | DBS on |  | DBS off | reposition |  |
| 17 | contacts | | 2 / 10 – 3 / 11 | |  | 1+,3-/ 10+,11- |  | 3 / 11 |  |  |
|  | voltage | | 5 / 5 V | |  | 5 / 6 V |  | 5 / 5 V |  |  |
|  | pulse width | | 90 μs | |  |  |  |  |  |  |
|  | frequency | | 140 Hz | |  |  |  |  |  |  |
|  | cycling on / off | | 1 / 5 min | | cycling off ^b^ | 1 / 5 min |  |  |  |  |
| 18 | contacts | | 2 / 10 | | 1+,2- / 9+,10- | 2 / 10 | 3 / 11 |  |  |  |
|  | voltage | | 4 / 4 – 5 / 5 V | |  | 4.5 / 4.5 V | 5 / 4 V |  |  |  |
|  | pulse width | | 90 μs | |  |  |  |  |  |  |
|  | frequency | | 140 Hz | |  |  |  |  |  |  |
|  | cycling on / off | | 1 / 5 min | |  |  |  |  |  |  |
|  | AED changes | |  | |  |  |  | ↓PER |  |  |
| 19 | contacts | | 2 / 10 | |  | 3 / 11 |  |  |  |  |
|  | voltage | | 5 / 5 V | |  | 4.5 / 3.5 V |  |  |  |  |
|  | pulse width | | 90 μs | |  |  |  |  |  |  |
|  | frequency | | 140 /s | |  |  |  |  |  |  |
|  | cycling on / off | | 1 / 5 min | |  |  |  |  |  |  |
|  | AED changes | |  | |  | -ESL, +LCM |  |  |  |  |
| 20 | contacts | | 1+,2- / 10+,9- | | 1+,2- / 10-,11+ | 1,2 / 8,9 |  |  |  |  |
|  | voltage | | 4 / 4 – 5 / 6 V | | 5 / 4 V | 5 / 5 V |  |  |  |  |
|  | pulse width | | 90 μs | |  |  |  |  |  |  |
|  | frequency | | 140 Hz | |  |  |  |  |  |  |
|  | cycling on / off | | 1 / 5 min | |  |  |  |  |  |  |
|  | AED changes | |  | |  |  |  | ↑LCM |  |  |
| 21 | contacts | | 3 / 11 | |  | 0+,2-,3- / 8+,10-,11- |  |  |  |  |
|  | voltage | | 5 / 5 V | | 5 / 6 V | 5 / 5 V |  |  |  |  |
|  | pulse width | | 90 μs | |  |  |  |  |  |  |
|  | frequency | | 140 Hz | |  |  |  |  |  |  |
|  | cycling on / off | | 1 / 5 min | |  |  |  |  |  |  |
|  | AED changes | |  | | ↓CLB |  |  |  |  |  |
| 22 | contacts | | 3 / 11 | |  |  | 0 / 8 | 2,3 / 10,11 | 2-,3+ /  10-,11+ |  |
|  | voltage | | 4 / 4 – 6 / 6 V | | 5 / 5 V | 6 / 6 V | 4 / 4 V | 4 / 4 V |  |  |
|  | pulse width | | 90 μs | |  |  |  | 90 μs |  |  |
|  | frequency | | 140 Hz | | 180 Hz |  | 140 Hz | 140 Hz |  |  |
|  | cycling on / off | | 1 / 5 min | |  |  |  | 1 / 5 min |  |  |
|  | AED changes | |  | |  | ↓VPA, +LCM |  |  | +PER |  |
|  | other | |  | |  |  |  | reposition |  |  |
| 23 | contacts | | 3 / 11 | |  | 3 / 11 |  |  |  |  |
|  | voltage | | 4 / 4 – 5 / 5 V | |  | 5 / 6 V |  |  |  |  |
|  | pulse width | | 90 μs | |  | 90 μs |  |  |  |  |
|  | frequency | | 140 Hz | |  | 140 Hz |  |  |  |  |
|  | cycling on / off | | 1 / 5 min | |  | 1 / 5 min |  |  |  |  |
|  | AED changes | |  | |  |  | -OXC, +ESL, ↑LCM, ↑LEV |  | +PER, ↓LEV |  |
|  | other | |  | | DBS off ^a^ |  |  |  |  |  |
| 24 | contacts | | 2 / 10 | |  | 2 / 11 | 2,3 / 10,11 |  |  |  |
|  | voltage | | 5 / 5 – 7 / 7 V | |  | 5 / 5 V | 4 / 4 |  |  |  |
|  | pulse width | | 90 μs | |  | 150 μs | 210 μs |  |  |  |
|  | frequency | | 140 Hz | | 180 Hz |  |  |  |  |  |
|  | cycling on / off | | 1 / 5 min | |  |  |  |  |  |  |
|  | AED changes | |  | | ↑CLB |  | -OXC, +LTG, ↑LCM |  |  |  |
|  | other | |  | |  | * | * |  |  |  |
| 25 | contacts | | 2+,3- / 10+,11- | |  | 3 / 11 |  |  |  |  |
|  | voltage | | 3.5 / 4 –  4.5 / 5 V | |  | 3.5 / 4.5 V |  |  |  |  |
|  | pulse width | | 90 μs | |  |  |  |  |  |  |
|  | frequency | | 140 Hz | |  |  |  |  |  |  |
|  | cycling on / off | | 1 / 5 min | |  |  |  |  |  |  |
|  | AED changes | |  | |  | ↑LCM, ↓TPR |  |  |  |  |
| 26 | contacts | | 2+,3- / 10-,11- – 3 / 11 | |  |  |  |  |  |  |
|  | voltage | | 5 / 5 V | |  |  |  |  |  |  |
|  | pulse width | | 90 μs | |  |  |  |  |  |  |
|  | frequency | | 140 Hz | |  |  |  |  |  |  |
|  | cycling on / off | | 1 / 5 min | |  |  |  |  |  |  |
| 27 | contacts | | 3 / 11 | | 3 / 10 | 3 / 11 |  |  |  |  |
|  | voltage | | 5 / 2.5 V | |  | 5 / 3.5 V |  |  |  |  |
|  | pulse width | | 90 μs | |  | 90 / 70 μs |  |  |  |  |
|  | frequency | | 140 Hz | |  | 130 Hz |  |  |  |  |
|  | cycling on / off | | 1 / 5 min | |  |  |  |  |  |  |
|  | other | |  | |  | cable revision |  |  |  |  |
