# Supplementary figures and images for: Improving the effectiveness of ANT DBS therapy for epilepsy with optimal current targeting
Source: Epilepsia Open. 2020 Aug 9;5(3):406–17. doi: 10.1002/epi4.12407 (PMC7469781; doi:10.1002/epi4.12407)

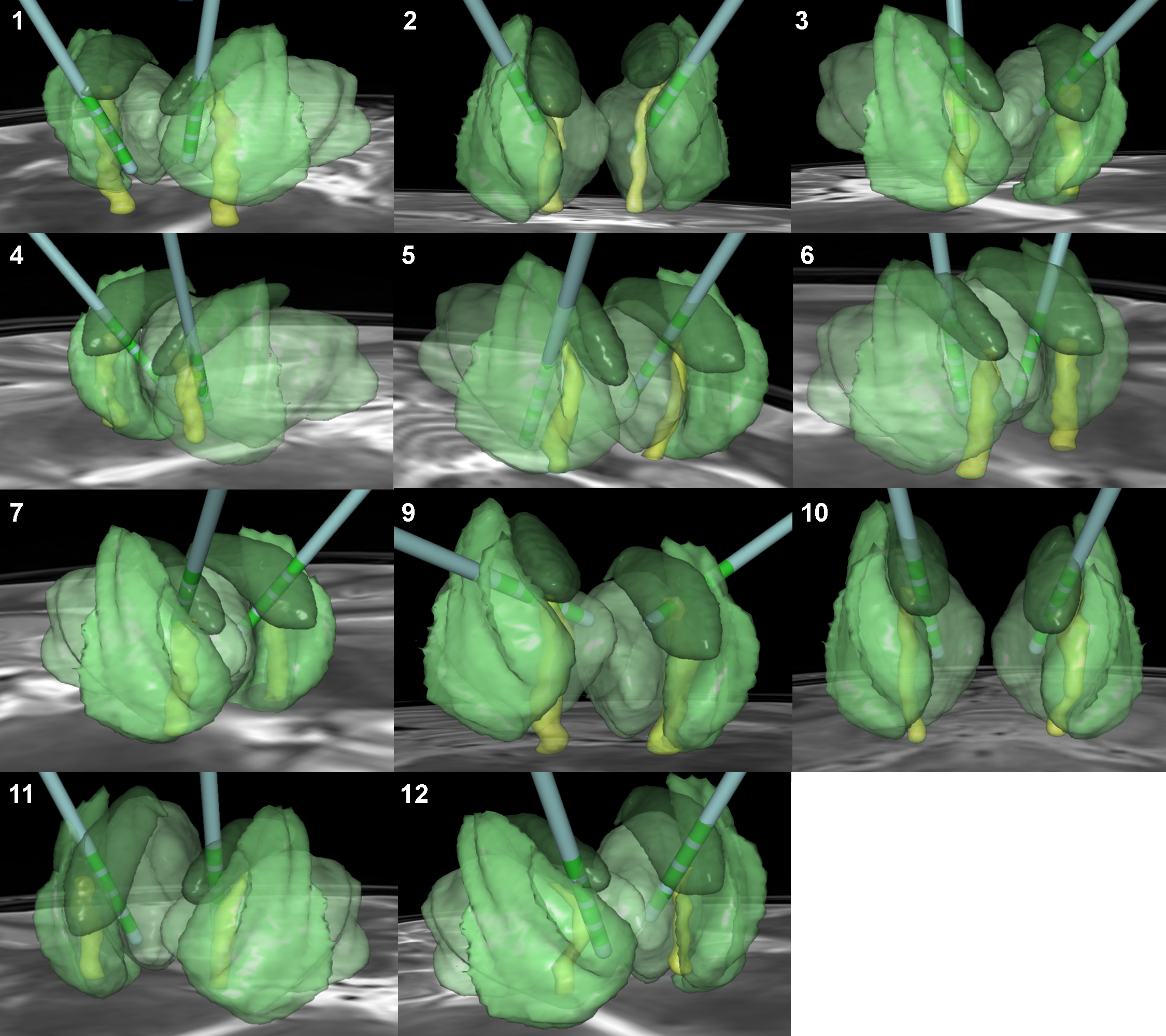

Supplement: Supplementary file 1 — Supplementary Material [file EPI4-5-406-s001.zip › epi412407-sup-0001-FigS1.1.tif]

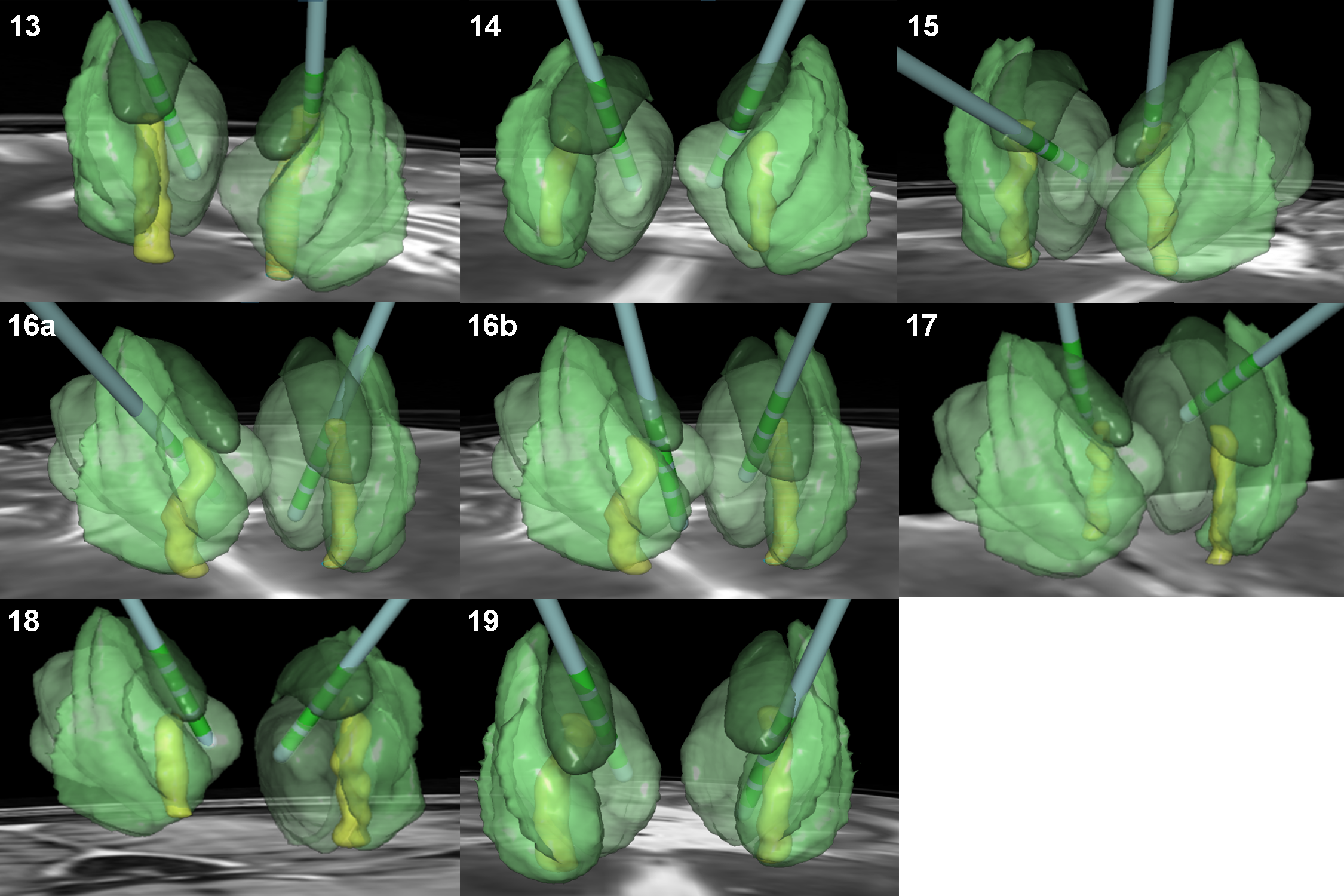

Supplement: Supplementary file 1 — Supplementary Material [file EPI4-5-406-s001.zip › epi412407-sup-0002-FigS1.2.tif]

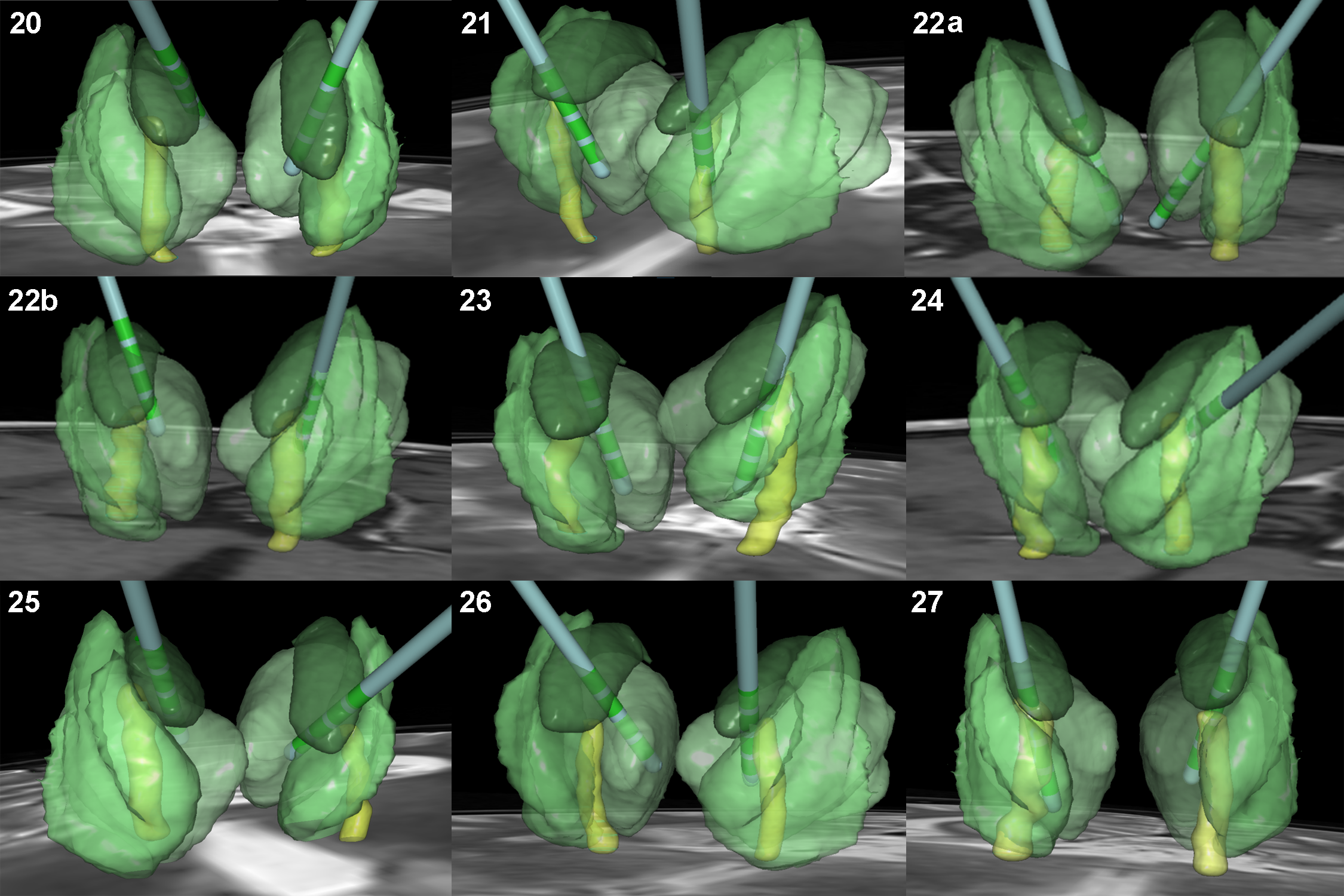

Supplement: Supplementary file 1 — Supplementary Material [file EPI4-5-406-s001.zip › epi412407-sup-0003-FigS1.3.tif]
